# Microglia TREM2: A Potential Role in the Mechanism of Action of Electroacupuncture in an Alzheimer's Disease Animal Model

**DOI:** 10.1155/2020/8867547

**Published:** 2020-09-04

**Authors:** Yujie Li, Jing Jiang, Qisheng Tang, Huiling Tian, Shun Wang, Zidong Wang, Hao Liu, Jiayi Yang, Jingyu Ren

**Affiliations:** Beijing University of Chinese Medicine, Beijing, China 100029

## Abstract

Alzheimer's disease (AD) is one of the most serious public health concerns facing the world. Its characteristic feature is neuroinflammation due to microglial activation. Electroacupuncture is one of the therapies employed to improve the condition of patients with AD, although its mechanism of action is still to be determined. Triggering receptor expressed on myeloid cells 2 (TREM2) is a microglia-specific receptor that is involved in regulating neuroinflammation in AD. In this study, we applied senescence-accelerated mouse-prone 8 mice as the AD animal model, used the Morris water maze, and applied hematoxylin and eosin staining, immunofluorescence double staining, and Western blotting, to explore the effects and potential mechanisms of action of electroacupuncture. In summary, this study suggested that electroacupuncture treatment could improve the learning and memory abilities (*p* < 0.05) and protect neurons. These effects result from acupuncture could upregulate TREM2 expression in the hippocampus (*p* < 0.01), which was essential for the anti-inflammatory effects in the AD animal model. However, further studies are needed to conclusively demonstrate the mechanism of action of electroacupuncture in AD.

## 1. Introduction

As the commonest cause of dementia, Alzheimer's disease (AD) is a growing global health concern with huge implications for individuals and society [1]. This neurodegenerative disease is characterized classically by two hallmark pathologies: *β*-amyloid plaque deposition and neurofibrillary tangles of hyperphosphorylated tau [2]. In recent years, there is increasing evidence for researchers to consider brain neuroinflammation as a new feature of AD [3], which is associated with microglial activation [4]. Microglia, as the resident immune cells of the central nervous system, play an important role in maintaining tissue homeostasis and contribute towards brain development under normal conditions [5]. However, when there is neuronal injury or other insults, depending on the type and magnitude of stimuli, microglia will be activated to secrete either proinflammatory factors that enhance cytotoxicity or anti-inflammatory neuroprotective factors that assist in wound healing and tissue repair [6]. Excessive microglial activation damages the surrounding healthy neural tissue, and the factors secreted by the dead or dying neurons in turn exacerbate the chronic activation of microglia, causing progressive loss of neurons [7]. Therefore, more researchers direct their energies into determining the mechanism of regulating microglial activation in AD.

Triggering receptor expressed on myeloid cells 2 (TREM2), as a new target in regulating microglial activation, is highly and exclusively expressed on microglia [8]. As a microglial surface receptor, TREM2 interacts with adaptor protein DAP12 (TYRO protein tyrosine kinase-binding protein (TYROBP), also known as DAP12) to initiate signal transduction pathways that promote microglial cell activation, phagocytosis, and microglial survival [9].

In order to improve the quality of life of patients with AD and delay the progression of the disease, acupuncture therapies are widely applied especially in China [10, 11]. Consequently, the mechanism of action of acupuncture in AD is to be elucidated to validate the observed therapeutic effects. Although some studies focused on the expression of TREM2 during the process of AD, there was little evidence to explain the mechanism of acupuncture modality from regulating the expression of TREM2 [7, 12]. In addition, in our previous studies [13, 14], we had found that acupuncture therapy could inhibit the expression of proinflammatory factors in the brain of AD animal models, such as IL-1 beta and NTF-alpha. Because of TREM2 in the microglia taking part in the regulation of these proinflammatory factors, we want to focus on the upstream of this process, to investigate whether acupuncture could regulate the expression of TREM2. Overall, these aspects of studies could provide a novel angle to demonstrate the acupuncture mechanism and scientific evidence to apply this modality in AD patients.

In this study, we investigated whether acupuncture therapy improved AD by regulating the expression of TREM2 on microglia using a recognized AD animal model.

## 2. Materials and Methods

### 2.1. Ethics Statement

The experimental protocol applied in this study was approved by the Ethics Committee for Animal Experimentation of Beijing University of Chinese Medicine (ID: BUCM-4-2018111701-4045). All procedures complied with the Animal Research: Reporting of In Vivo Experiments (ARRIVE) guidelines and were performed according to the guidelines of the National Institutes for Animal Research. All researchers in this study were certified by the Animal Experimentation Center of Beijing University of Chinese Medicine.

### 2.2. Animals

Eight-month-old senescence-accelerated mouse-prone 8 (SAMP8) mice [15] and senescence-accelerated mouse-resistant 1 (SAMR1) mice weighing 30.0 ± 2.0 g were purchased from the Experimental Animal Center of the First Teaching Hospital of Tianjin University of Traditional Chinese Medicine (Animal Lot: SCXK (Jing) 2014-0003). The animals were housed in the Animal Experimentation Center of Beijing University of Chinese Medicine at controlled temperature (24 ± 2°C) and under a 12 h dark/light cycle, with sterile drinking water and a standard pellet diet available ad libitum. All mice were acclimatized to the environment for five days prior to experimentation.

### 2.3. Grouping and Intervention

Ten SAMR1 male mice were placed in the control group, and 30 SAMP8 male mice were randomly divided into three experimental groups of 10 mice each: the AD model control group (AD group), drug group, and electroacupuncture group (EA group). In the drug group, donepezil hydrochloride tablets (Eisai China Inc., H20050978) were dissolved in distilled water and delivered to the mice by oral gavage at a dose of 0.65 μg/g. In the EA group, mice were immobilized in mouse bags. *Baihui* (GV20) and *Yintang* (GV29) were chosen for electroacupuncture for 15 min per day, with transverse puncturing at a depth of 4–5 mm using disposable sterile acupuncture needles (0.25 mm × 13 mm) (Beijing Zhongyan Taihe Medicine Company). The needles were taped and connected to the HANS-LH202 electroacupuncture device (Peking University Institute of Science Nerve and Beijing Hua Wei Industrial Development Company, Beijing, China), with the sparse wave at 2 Hz, 2 V, and 0.1 mA. The mice in the control group, AD group, and drug group received the same 15-minute restriction as those in the EA group.

### 2.4. Morris Water Maze Test

After the 15-day intervention, mice from each group were evaluated in the Morris water maze [16]. The Morris water maze consisted of a circular tank (diameter, 120 cm; height, 50 cm) filled with opaque water, rendered with milk powder to a depth of 30 cm. A video camera (TOTA-450d, Japan) fixed to the ceiling and connected to a video recorder with an automated tracking system (China Daheng Group, China) was used to collect data. A removable platform (diameter, 9.5 cm; height, 30 cm) was placed inside the pool (at quadrant III). The pool area was conceptually divided into four quadrants (I, II, III, and IV) of equal size. Visual cues of different shapes were placed on the tank wall of each quadrant in plain sight of the mice.

#### 2.4.1. Hidden Platform Trial

This trial was applied for testing the spatial learning ability of the mice. All surviving mice were trained in the standard Morris water maze with a hidden platform. Quadrants I and II were selected as entry points. Each mouse was released from the two points and had 60 s to search for the hidden platform. Two trials per day for 5 consecutive days were performed, with the visual cues kept constant.

#### 2.4.2. Probe Trial

This trial was used for testing the spatial memory ability of the mice. The platform was removed the day after the hidden platform trial. Each mouse was placed in the pool once for 60 s, starting from the same initial location used in the hidden platform trial. The crossing times of the platform were recorded during the test.

### 2.5. Hematoxylin and Eosin (HE) Staining

To observe the neurons in the dentate gyrus of the hippocampus in each group, three mice were randomly chosen from each group after the Morris water maze tests, anesthetized, and had their brains removed and subjected to HE staining. Tissues were dehydrated, paraffin-embedded, and sliced (thickness, 10 mm/slice), followed by dewaxing three times in xylene for 5 min each, and then placed in anhydrous ethanol for 5 min, followed by 90% ethanol, 70% ethanol, and distilled water, each for 2 min, and then made to undergo HE staining. Thereafter, sections were dehydrated through increasing concentrations of ethanol and xylene. The dentate gyrus of the hippocampus in each specimen was viewed under a microscope (Olympus, BX53; magnification, ×40).

### 2.6. Immunofluorescence Double Staining

Immunofluorescence double staining was used to detect the coexpression of microglia and TREM2 expression in the dentate gyrus of the hippocampus. Sections were dewaxed and hydrated, placed in Triton X-100 (mass fraction 0.5%) for 10 min, blocked with bovine serum albumin (mass fraction 2%), and rested for 1 h at room temperature. Antibody TREM2 (Proteintech, USA; 1 : 150) was mixed with antibody Iba-1 (Proteintech, USA; 1 : 150) and incubated on sections overnight at 4°C and rinsed with phosphate-buffered saline (PBS) three times. The FITC fluorescent-labeled secondary antibody (1 : 200) was mixed with the TRITC fluorescent-labeled secondary antibody (1 : 200), incubated on the slices at room temperature for 2 h, and then rinsed with PBS. The slices were scanned and imaged by the digital pathological slice scanner (NanoZoomer S210, Hamamatsu, C13239-01) after drying and then analyzed.

### 2.7. Western Blotting

To test for the expression of TREM2 protein in the hippocampus, seven mice in each group were sacrificed under anesthesia to harvest their hippocampi. After protein extraction, SDS- polyacrylamide gel electrophoresis was performed using a 10% separating gel and a 5% stacking gel. Proteins were then transferred to a 0.45 μm polyvinylidene fluoride membrane. Membrane blocking was performed using 5% nonfat milk in Tris-buffered saline supplemented with 0.1% Tween 20. Membranes were incubated in the primary antibody (Proteintech, USA; TREM2, mouse, 1 : 1000) at 4°C overnight. The secondary antibody (1 : 2000) was added before shaking and incubating at room temperature for 1 h. The HRP-ECL luminous liquid was added, and X-ray film exposure was completed in a dark room following development and fixing. After calibrating the control markers, the scanning and analyses were performed by The Discovery Series Quantity One, version 4.6, and the relative and standardized levels of caspase-3 and tau expression were compared in each group.

### 2.8. Statistical Analyses

Statistical analyses were performed using the SPSS software, version 25.0 (SPSS, Inc., Chicago, IL, USA), and data was expressed as the mean ± standard deviation. Multivariate analysis of variance (ANOVA) with repeated measures was used for the general linear model using the SPSS software, and pairwise comparison was used for the different groups and different measurement times. First, Mauchly's test of sphericity was used to assess whether there were relations among the repeatedly measured data. For results with *p* ≤ 0.05, multivariate ANOVA or Greenhouse-Geisser correction was performed. The effect of treatment was evaluated by estimating the between-subject variance. Repeated measurement effect or its interactive effect with the treated group was evaluated by estimating the within-subject variance. The Bonferroni test was used to perform pairwise comparisons of the repeatedly measured data in different measurement times in each treated group. Multivariate ANOVA was used for pairwise comparison of data in different groups for each measurement time. One-way ANOVA was used after the normal distribution and homogeneity of variance were confirmed for the other dates.

## 3. Results

### 3.1. EA Could Improve Learning and Memory Abilities in the AD Animal Model

**Figure 1:**
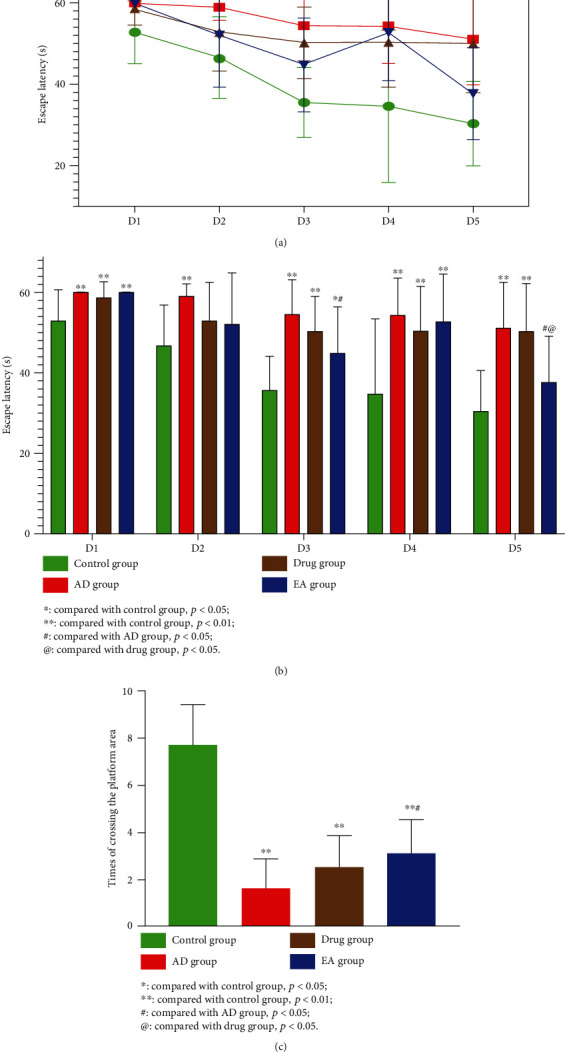
Results of the Morris water maze tests (10 mice of each group). (a, b) Results of the hidden platform trial, showing the spatial learning abilities of the mice in each group, the EA group performing better than the AD group on the third day and fifth day (third day, *p* = 0.029 < 0.05; fifth day, *p* = 0.011 < 0.05) and better than the drug group on the fifth day (*p* = 0.019 < 0.05). (c) Results of the probe trial, showing the spatial memory abilities of the mice in each group, the EA group performing better than the AD group (*p* = 0.012 < 0.05).

The results of the Morris water maze tests are presented in Figure 1. From Figure 1(a), we observed that the escape time of each group shortened according to the training days accumulated. Interestingly, on the fourth day of the hidden platform test, the escape times of the EA group mice were longer than those of the third day, and on the fifth day (last day of the training), this data significantly decreased, even shorter than the third day. After the fourth day of the hidden platform trial, we reexamined the device, discovered the fault, and applied corrective measures. Thereafter, we evaluated the mice of the EA group and considered whether 3 consecutive days of training had caused excessive fatigue, preventing them from finding the platform. Therefore, on the fifth day of the hidden platform trial, we rested the EA group mice, only testing them after the mice from the three experimental groups had been tested. From the obtained results, our hypothesis proved to be correct.

From Figure 1(b), we observed that the escape times of the AD group mice were significantly longer than those of the control group mice for each day (*p* = 0.001 < 0.01), which meant that the spatial learning ability of SAMP8 mice was significantly lower. However, on the third and fifth days, the escape times of the EA group mice were significantly shortened (third day, *p* = 0.029 < 0.05; fifth day, *p* = 0.011 < 0.05), even shorter than those of the drug group on the last day (*p* = 0.019 < 0.05). These results illustrated that electroacupuncture treatment may improve the spatial ability of SAMP8 mice.

Figure 1(c) shows the platform quadrant crossing times for each group. There was a significant difference between the control group mice and the mice from the three experimental groups (*p* = 0.001 < 0.01). Moreover, the EA group mice were more than the AD group mice in this data (*p* = 0.012 < 0.05), which implied that spatial memory ability was also improved by electroacupuncture treatment.

### 3.2. EA Could Be Neuroprotective in the Hippocampus of the AD Animal Model

**Figure 2:**
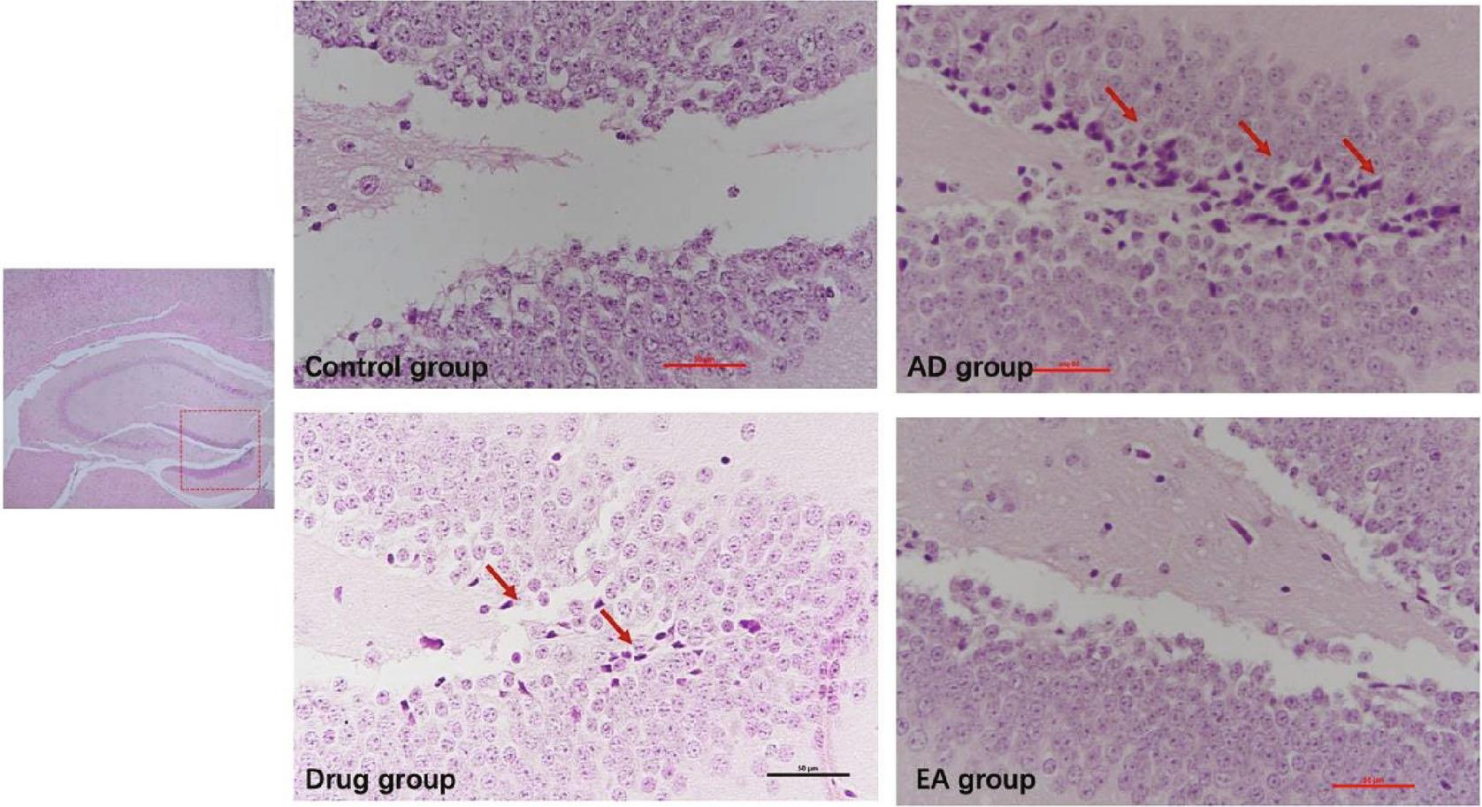
Results of HE staining in each group (3 samples of each group). In this study, we focused on the dentate gyrus zone in the hippocampus, the zone that was most related with learning and memory abilities. The red arrows point at the most neurons that lost their function in the AD group; compared with the AD group, the EA group observed less neuron apoptosis in the hippocampus.

Figure 2 shows the HE staining results of the dentate gyri of the hippocampi. The control group mice demonstrated clear-dyed neurons aligned in neat rows, with round nuclei and distinct kernels in the dentate gyri. Conversely, neurons tended to be scattered and irregular, with indistinct kernels and nuclear pyknosis in the dentate gyri of the AD group mice. Subjectively, neurons of the mice in the drug and EA groups were more neatly arranged in rows and clearer in structure with less nuclear condensation, as compared to those of the AD group mice. Moreover, tissue samples from the EA group mice appeared to be most similar to those from the control group. These demonstrated that electroacupuncture may be neuroprotective to some extent.

### 3.3. EA Could Upregulate the Expression of TREM2 on Microglia

To investigate the potential neuroprotective effect of electroacupuncture in the AD animal model from the viewpoint of microglia regulating neuroinflammation and test the expression of TREM2 protein, we applied immunofluorescence double staining and Western blotting.

**Figure 3:**
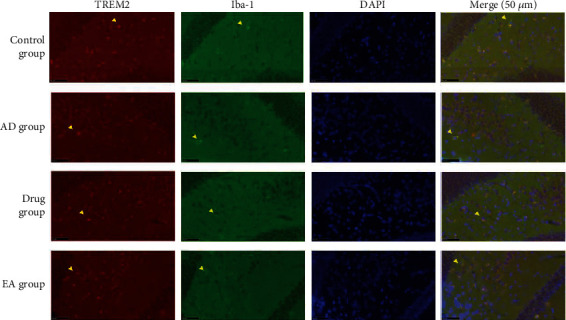
Results of immunofluorescence double staining in each group (magnification, ×400; 3 samples of each group). Red light revealing the expression of TREM2 on microglia, green light showing the expression of Iba-1 on microglia, and blue light showing the DNA of the nucleus in the brain. The merge showed the coexpression of TREM2 and Iba-1. The yellow arrows of each group pointed the expression of TREM2 and Iba-1 and the coexpression of TREM2 and Iba-1. We found that both TREM2 and Iba-1 were expressed on the membranes of microglia.

Red light was used to label TREM2 and green light to label microglia (Iba-1) (Figure 3). It was observed that TREM2 proteins were mainly expressed on the membranes of microglia and in the dentate gyri of the hippocampi; the expression of TREM2 in the control group mice was higher than that of the AD group mice.

**Figure 4:**
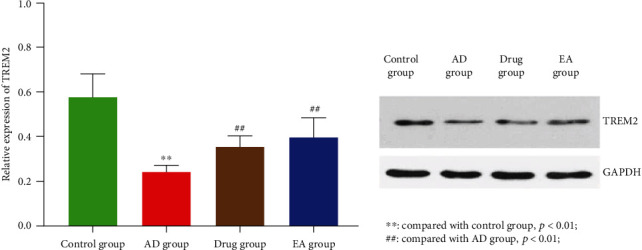
Results of the Western blotting test (in the hippocampus; 7 samples of each group). The relative expression of TREM2 for each mouse group revealing that electroacupuncture may significantly increase the expression of TREM2 in the hippocampus (*p* = 0.001 < 0.01).

Figure 4 shows the concentration of TREM2 proteins in the hippocampi by Western blot. The concentration of TREM2 in the hippocampi of the control group mice was found to be higher than that of the AD group mice (*p* = 0.001 < 0.01). Moreover, the concentrations of TREM2 in the hippocampi of the EA group and drug group mice were also higher than that of the AD group mice (*p* = 0.001 < 0.01, respectively). There were only numerical advantages and no significant differences in the concentrations of TREM2 in the EA group and drug group mice.

## 4. Discussion

Although research into the clinical and pathological features of AD spans nearly 120 years, there are still no effective treatment modalities to modify or reverse this disease [17]. Therefore, management of the risk factors associated with the disease and improving cognitive impairment or slowing the progression of the disease are the current strategies [18].

Acupuncture therapy, a treatment modality of Chinese medicine, is widely recognized and used in many countries and regions in the world. At present, acupuncture therapy is applied for improving a variety of central nervous system diseases: dementia [19, 20], stroke [21, 22], Parkinson's disease [23–26], spinal cord injury [27, 28], and so on [29, 30]. Clinical research in AD found this therapy to be safe, well tolerated, and effective in improving cognitive function [10].

In this study, it was demonstrated that electroacupuncture may improve spatial learning and memory abilities and be neuroprotective in the hippocampus. These results were consistent with our previous research [31, 32]. Although the escape time of the EA group mice during the hidden platform trial in the Morris water maze on the fourth day was significantly higher than that on the day before, they performed the best among the three experimental SAMP8 mouse groups on the fifth day.

The aim of this study was not only to determine the effects of electroacupuncture but also to investigate its potential mechanism of action. According to recent studies and available evidence, we considered that neuroinflammation activated by brain microglia may be “the third pathological feature” of AD [33, 34]. The magnitude of microglial activation depends on extrinsic and intrinsic conditions, for example, the type of insult, potency of the stimulus, distance from the stimulus, and immediate microenvironment [35]. Studies have implicated that the amyloid *β*-protein in the pathogenesis of AD was associated with the endogenous ligand of TREM2 [36, 37]. Moreover, current studies utilizing AD mouse models as well as human tissue have found that loss of TREM2 prevented microglia from accumulating around amyloid plaques, causing deficits in the barrier that limited neuronal injury [9]. Therefore, the expression of TREM2 on microglia could proliferate the progression of AD and be used as a target protein to demonstrate the mechanism of action of acupuncture. Another study attempting to elucidate the mechanism of action of acupuncture therapy in improving cerebral ischemia showed that TREM2 could be a potential target in EA treatment for attenuating inflammatory injury following cerebral ischemia/reperfusion [38]. However, since there were few studies evaluating the role of TREM2 expression in the mechanism of action of acupuncture therapy in AD, this study attempted to investigate the presence of any association.

In our study, immunofluorescence double staining and Western blotting were applied to illustrate the changes in the expression of TREM2 on microglia in the hippocampus of the AD animal model after electroacupuncture treatment. The results demonstrated that electroacupuncture might be neuroprotective in the hippocampus by upregulating the expression of TREM2 on microglia. In this study, we only focused on the expression of the core protein TREM2 in the hippocampus. Based on our performed studies, we found that the upregulation of TREM2 might induce the downregulation of some proinflammatory factors, such as IL-1*β* and TNF-*α*. However, as we all know, the neuroinflammation reaction activated by microglia is a complex process, which is associated with many kinds of proteins, cytokines, and proinflammatory factors [39]. Therefore, there must be some other proteins taking part in the process, which we could not examine in this study. So, in further studies, we should not only try to explore the regulation of TREM2 but also try to consider the other relative proteins, especially the downstream protein of TREM2.

## 5. Conclusion

In summary, this study suggested that electroacupuncture treatment could upregulate TREM2 expression, which is essential for the anti-inflammatory effects to protect the neurons and improvement in the learning and memory abilities in the AD animal model. However, more studies are needed to clearly elucidate the mechanism of action of electroacupuncture in AD.

## Data Availability

The data used to support the findings of this study are included within the article.
